# A Brief Review of the Microweiseinae (Coleoptera: Coccinellidae) of the Indian Region, Including Description of a New Species †

**DOI:** 10.3390/insects15110874

**Published:** 2024-11-07

**Authors:** Janakiraman Poorani

**Affiliations:** ICAR-National Research Centre for Banana, Thogamalai Road, Thayanur Post, Tiruchirappalli 620102, India; pooranij@gmail.com

**Keywords:** Coccinelloidea, Indian Subcontinent, pictorial guide, biological control

## Abstract

Indian species of Microweiseinae are reviewed, comprising eight species (five species of *Serangium* Blackburn, one species of *Microserangium* Miyatake, and two species of *Scymnomorphus* Weise, including one new species from the southern Indian state of Kerala, *Scymnomorphus ochraceus* sp. n.). Illustrated diagnostic accounts are given for all species with details of distribution, biology, and host insects, wherever they are known. Immature stages are illustrated for *Serangium parcesetosum* Sicard and *Serangium chapini* (Kapur). An updated checklist of the Microweiseinae of India is given. The need for focused collections and systematic studies to unearth the hidden diversity of Indian Microweiseinae is discussed, and comments on their poor utilization in applied biological control in India are made.

## Simple Summary

The family Coccinellidae (Coleoptera) has only three recognized subfamilies, Coccinellinae, Monocoryninae, and Microweiseinae, of which Microweiseinae is poorly represented in the Indian region mainly due to the lack of focused collections. Of the four currently known tribes in Microweiseinae, only Serangiini and Microweiseini are represented in the Indian Subcontinent. Diagnostic accounts of Serangiini and Microweiseini of mainland India are presented here with a key to genera and species, with a description of a new species of *Scymnomorphus* Weise and an updated checklist of Microweiseinae of this region.

## 1. Introduction

The family Coccinellidae (Coleoptera: Coccinelloidea) has only three recognized subfamilies, Coccinellinae, Monocoryninae, and Microweiseinae. Members of the subfamily Microweiseinae are hard to spot and among the smallest and least recognizable Coccinellidae, and the small, pubescent body forms of Microweiseinae more closely resemble some Anamorphidae, Mycetaeidae, and Corylophidae than “true” lady beetles. The Microweiseinae often have the head withdrawn into the pronotum or deflexed and held closely against the ventral side, and also lack the familiar aposematic coloration of larger coccinellids [1]. Species of the genera *Serangium* Blackburn, 1866 [2] and *Delphastus* Casey, 1899 [3] (Serangiini) are well-known and apparently specific predators of whiteflies (Hemiptera: Sternorrhyncha: Aleyrodidae), including major pest whiteflies on various crops and are widely used for the management of whiteflies in glasshouses [4,5,6,7,8]. *Serangium parcesetosum* Sicard and *S. montazerii* Fürsch, two of the most widely studied and utilized species of Serangiini in applied biological control, are naturally distributed in India [9]. Members of Microweiseini are predators of armoured scale insects (Hemiptera: Diaspididae) [8,9,10,11,12,13,14,15,16].

Escalona and Ślipiński [1] reviewed the subfamily Microweiseinae of the world and studied the phylogenetic relationships of all the known genera and tribes of Microweiseinae. They recognized three tribes: Carinodulini Gordon et al., Serangiini Pope, and Microweiseini Leng. They provided diagnostic accounts of the tribes and genera with illustrations and a revised phylogenetic analysis of the tribes with an updated key. Recent molecular studies on the superfamily Cucujoidea by Robertson et al. [17] place Microweiseinae as an intermediate clade between the remaining Coccinellidae and Endomychidae *sensu lato*. Szawaryn et al. [18] added a fourth tribe, Madeirodulini, to Microweiseinae and provided a revised key to the tribes of Microweiseinae. The subfamily contains 23 genera and around 150 described species, representing about 3.5% of the Coccinellidae species, distributed mostly in tropical and subtropical ecosystems [1,18]. The tribe Serangiini comprises the oldest representatives of this group known from Eocene Baltic amber [19].

Only three genera of Microweiseinae, *Serangium* and *Microserangium* Miyatake, 1961 [20] (Tribe Serangiini) and *Scymnomorphus* Weise, 1897 [21] (Tribe Microweiseini), are known from India at present. This group is not commonly collected, and specimens are not found in institutional collections in India. Hence, this work is based on limited study material as systematic collection efforts are lacking for Microweiseinae, and representative collections from all parts of the Indian region are not available for examination. A brief, illustrated review of the Indian species of Microweiseinae is presented here, and one new species of *Scymnomorphus* is described from Kerala, South India. Life stages are illustrated for two species of *Serangium.* An updated checklist of the Indian Microweiseinae is given.

## 2. Materials and Methods

Specimens examined for this study are deposited in the following collections: BMNH: Natural History Museum, London; ICAR-NBAIR: ICAR-National Bureau of Agriculturally Important Insects, Bengaluru; ICAR-NRCB: ICAR-National Research Centre for Banana, Tiruchirappalli.

The genitalia were dissected, cleared in 10% NaOH solution, and transferred to glycerol on a slide for imaging and further studies. After examination, the genitalia were transferred to microvials and pinned beneath the specimen. Imaging was carried out using a Leica M205A stereo microscope (M/s. Leica Mikrosysteme Vertrieb GmbH, Wetzlar, Germany) with a Leica DMC4500 digital camera attachment and touched up for clarity using paint.net 4.3.12.

Micro and macro photographs of the immature stages were taken at various points in time and during laboratory rearing. The terminology used in this paper follows Ślipiński [22] and Lawrence et al. [23].

## 3. Results

### 3.1. Taxonomic Accounts

Key to the tribes of Microweiseinae of the Indian region (slightly modified from Escalona and Ślipiński [1])

Dorsal pubescence sparse, confined to the head, pronotum, and margins of elytra. Prosternum raised in the form of a large triangular plate, its anterior margin closes with anterior margin of clypeus in repose; ventral side of the body, including epipleura deeply foveate, to receive folded legs; abdomen with 5 ventrites, ventrites 1 and 2 never fused, ventrite 5, as long as ventrites 2–4 combined; antenna composed of eight or nine antennomeres, terminal antennomere forming a flattened club………….…………….…Serangiini

–Dorsal pubescence prominent, distributed all over the dorsum. Prosternum variable, flat, strongly reduced, or variously lobed anteriorly, partially or almost completely concealing mouthparts; ventral side of the body including epipleura without foveae to receive folded legs; abdomen with five or six ventrites, ventrites 1 and 2 incompletely or completely fused, ventrite 5 almost always short; antenna composed of eight to ten antennomeres, terminal one to three antennomeres forming a club………….…………….…Microweiseini

### 3.2. Tribe Serangiini

**Figure 1:**
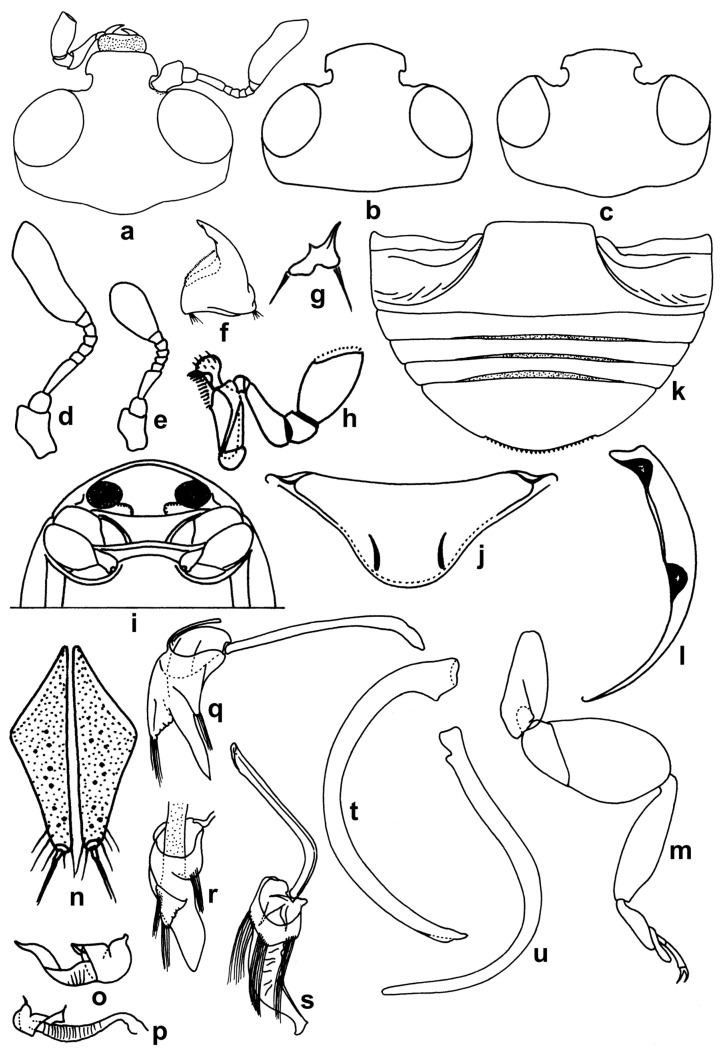
Diagnostic characters of Serangiini: (**a**–**c**) head; (**d**,**e**) antenna; (**f**,**g**) mandible; (**h**) maxilla; (**i**,**j**) prosternum; (**k**) abdomen; (**l**) epipleuron; (**m**) hindleg in *Serangium parcesetosum*; (**n**) coxites; (**o**,**p**) spermatheca; (**q**–**s**) tegmen; (**t**,**u**) penis.

**Diagnosis.** Body hemispherical to slightly longer than broad, compact and convex; dorsal side shiny, pubescence restricted to pronotum and margins of elytra. Head (Figure 1a–c) slightly prolonged anterior to antennal sockets, emarginate around antennal insertions; eyes coarsely faceted. Antennae (Figure 1d,e) with 8–9 antennomeres, with terminal antennomere forming an elongate, flattened club. Mandibles (Figure 1f,g) apically simple. Maxilla with terminal palpomere barrel-shaped to elongate conical (Figure 1h). Prosternum (Figure 1i) triangular, anteriorly lobed, concealing mouthparts and notched on either side to receive retracted antennae at rest, rarely with a pair of carinae (Figure 1j). Epipleura (Figure 1l) foveate to receive meso- and metafemoral apices. Legs with broad and flat femora (Figure 1m), concealing tibia when retracted; tarsi cryptotetramerous or trimerous. Abdomen (Figure 1k) with five visible sternites, abdominal postcoxal lines incomplete. Female genitalia with elongate triangular coxites (Figure 1n), spermatheca characteristic (Figure 1o,p). Penis guide of male genitalia (Figure 1q–u) asymmetrical, with reduced parameres having elongate apical setae.

The genera of Serangiini were reviewed by Miyatake [24,25]. There are five genera worldwide, of which *Serangium* Blackburn and *Microserangium* Chapin, are known from this region. The species of this tribe are specific predators of whiteflies, and rarely associated with scales.

**Genus *Microserangium* Miyatake**

*Microserangium* Miyatake, 1961: 37 [20]. Type species: *Microserangium shikokense* Miyatake, 1961 [20], by original designation.–Sasaji, 1971: 61 [26]; Miyatake, 1994: 241 [25]; Poorani, 2000: 45 [27]; Ślipiński and Burckhardt, 2006: 49 [28] (synonymized with *Serangiella* Chapin, 1940 [29]). Wang et al. 2013: 1 [30] (removal from synonymy with *Serangiella*).

*Serangiella* Chapin, 1940: 271 [29]. Unavailable name: Wang et al. 2013: 1 [30].

**Figure 2:**
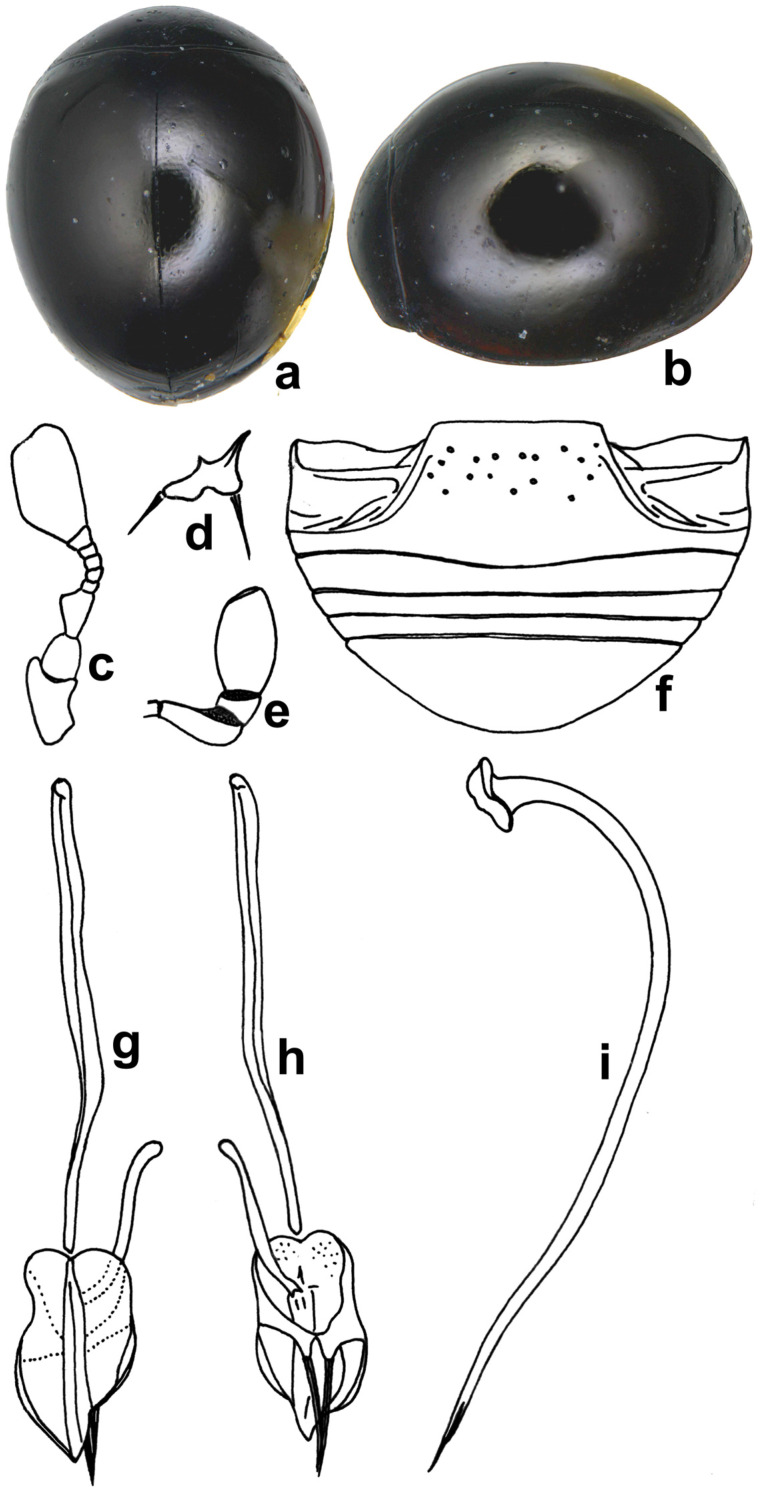
*Microserangium brunneonigrum* Poorani: (**a**) dorsal view; (**b**) lateral view; (**c**) antenna; (**d**) mandible; (**e**) terminal maxillary palpomere; (**f**) abdomen; (**g**–**i**) male genitalia: (**g**) tegmen, inner view; (**h**) tegmen, ventral view; (**i**) penis.

**Diagnosis.** *Microserangium* can be distinguished from *Serangium* by its usually much smaller size, reduced mandibles with external border deeply emarginate (Figure 2d), antenna with nine antennomeres, antennomere 3 short, asymmetrical and subtriangular (Figure 2c).

**Distribution.** Vietnam, India, Sri Lanka, China, and Japan [1].

**Notes.** Wang et al. [30] reviewed the Chinese species of *Microserangium* and commented on the status of *Serangiella* Chapin, an unavailable name which was treated as a senior synonym of *Microserangium* by Escalona and Ślipiński [1].

**Indian species.** At present, only *Microserangium brunneonigrum* Poorani is known from India.

***Microserangium brunneonigrum* Poorani**

(Figure 2)

*Microserangium brunneonigrum* Poorani, 2000: 45 [27].

*Serangiella brunneonigra*: Escalona and Ślipiński 2012: 145 [1].

**Diagnosis**: Length: l.11–1.17 mm; width: 0.93–1.00 mm. Form small, slightly longer than broad; dorsum strongly convex, hemispherical, pubescence confined to pronotum and lateral and basal margins of elytra. Dorsal side, dark brown to dark piceous to black, shiny (Figure 2a,b); pronotum, sutural line, and external borders of elytra darker than rest of body; ventral side lighter castaneous, except antennae, mouthparts, and tarsi yellowish brown. Legs with angulate tibiae and trimerous tarsi. Antenna (Figure 2c) with nine antennomeres, antennomere 3 elongate, subtriangular, fourth to seventh quadrate, eighth transverse, terminal antennomere forming an enlarged, angular club. Abdominal postcoxal line (Figure 2f) incomplete. Male genitalia (Figure 2g–i) as illustrated.

**Distribution.** India (Tamil Nadu).

**Prey/associated habitat.** Associated with unidentified whiteflies infesting *Homonoia riparia* and *Ficus hispida* [27].

**Seasonal occurrence.** Collected in July (label data).

**Notes.** This is the only species of *Microserangium* currently known from India [27], and a few adults seen in other collections are not available for further study.

**Genus *Serangium* Blackburn**

*Serangium* Blackburn, 1889: 209 [2]. Type species: *Serangium mysticum* Blackburn, 1889 [2], by monotypy.–Sicard, 1909: 151 [31]; Chapin, 1940: 268 [29]; Sasaji, 1967: 8 [32], 1971: 52 [26]; Miyatake, 1994: 239 [25]; Ślipiński and Burckhardt, 2006: 39 [28]; Ślipiński, 2007: 53 [21]; Wang et al. 2011: 1 [33].

*Semichnoodes* Weise, 1892: 15 [34]. Type species: *Semichnoodes kunowi* Weise, 1892 [33], by monotypy. Synonymized by Weise, 1908: 13 [35].

*Catana* Chapin, 1940: 266 [29]. Type species: *Catana clauseni* Chapin, 1940 [29], by original designation.–Sasaji, 1967: 8 [32]; Gordon, 1977: 209 [14]; Miyatake, 1994: 240 [25]. Synonymized by Ślipiński and Burckhardt, 2006: 39 [28].

*Catanella* Miyatake, 1961: 136 [23]. Type species: *Catanella formosana* Miyatake, 1961 [24], by original designation.–Sasaji, 1967: 10 [32]; Miyatake, 1994: 242 [25]. Synonymized by Ślipiński and Burckhardt, 2006: 39 [28]. Treated as a valid genus by Wang et al. 2011: 35 [33].

**Diagnosis.** Form circular to short oval, dorsum hemispherical, strongly convex, and shiny. Mandible distinct with conspicuous prostheca. Pronotum, lateral and anterior margins of elytra with fine, sparse pubescence. Antennae with nine antennomeres, third antennomere cylindrical/parallel-sided, last antennomere forming an elongate, flattened club, spatulate to pear-shaped. Terminal maxillary palpomere somewhat barrel-shaped, apex obliquely truncate. Prosternum strongly elevated and prominent anteriorly, forming a broad triangular shield covering mouthparts, anteriorly truncate and laterally notched to accommodate antennal club. Legs with broad femora, middle and hind tibiae not angulated externally; tarsi three- or four-segmented.

**Distribution.** Old World [1].

**Indian species.** Five species, namely, *S. montazerii* Fürsch, *S. parcesetosum* Sicard, *Serangium chapini* (Kapur), *S. clauseni* (Chapin), and *S. serratum* Poorani, are known from this region. The first two have been successfully introduced in other countries of the world to control whiteflies.

**Figure 3:**
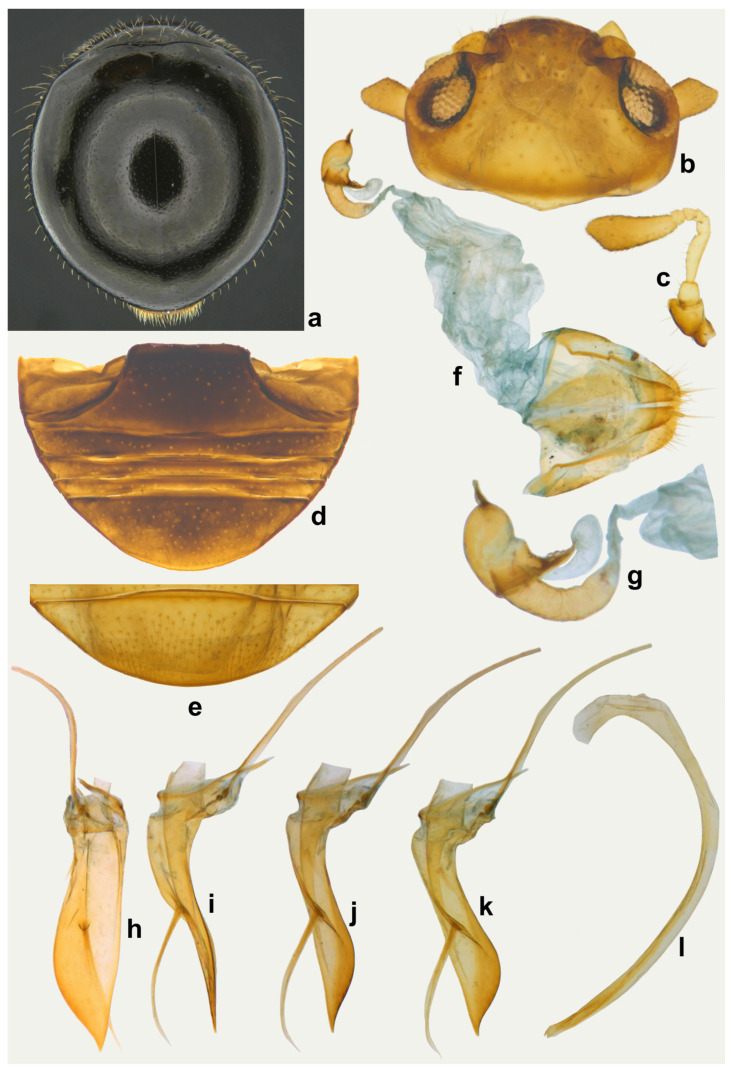
*Serangium chapini* (Kapur): (**a**) dorsal view; (**b**) head; (**c**) antenna; (**d**) abdomen, female; (**e**) terminal abdominal ventrites, male; (**f**) female genitalia; (**g**) spermatheca; (**h**–**l**) male genitalia: (**h**) tegmen, ventral view; (**i**–**k**) tegmen, lateral view; (**l**) penis.

**Figure 4:**
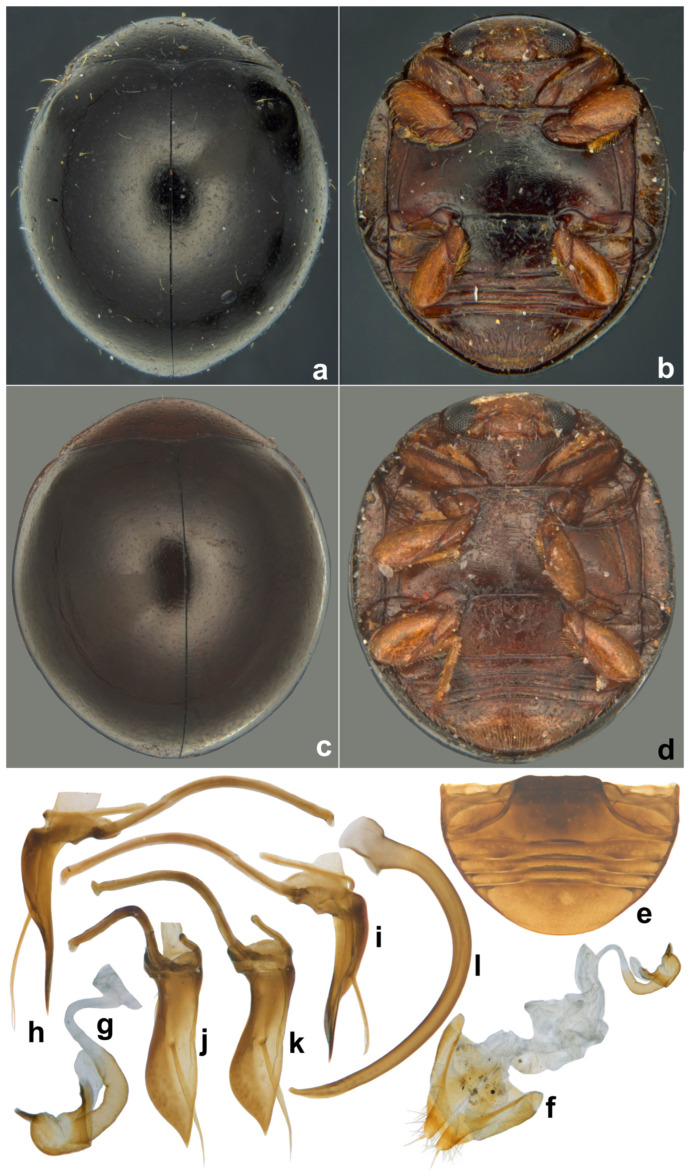
*Serangium chapini* (Kapur): (**a**,**c**) variants, dorsal view; (**b**,**d**) ventral view of variants; (**e**) abdomen, female; (**f**) female genitalia; (**g**) spermatheca; (**h**–**l**) male genitalia: (**h**,**i**) tegmen, lateral view; (**j**,**k**) tegmen, ventral view; (**l**) penis.

**Figure 5:**
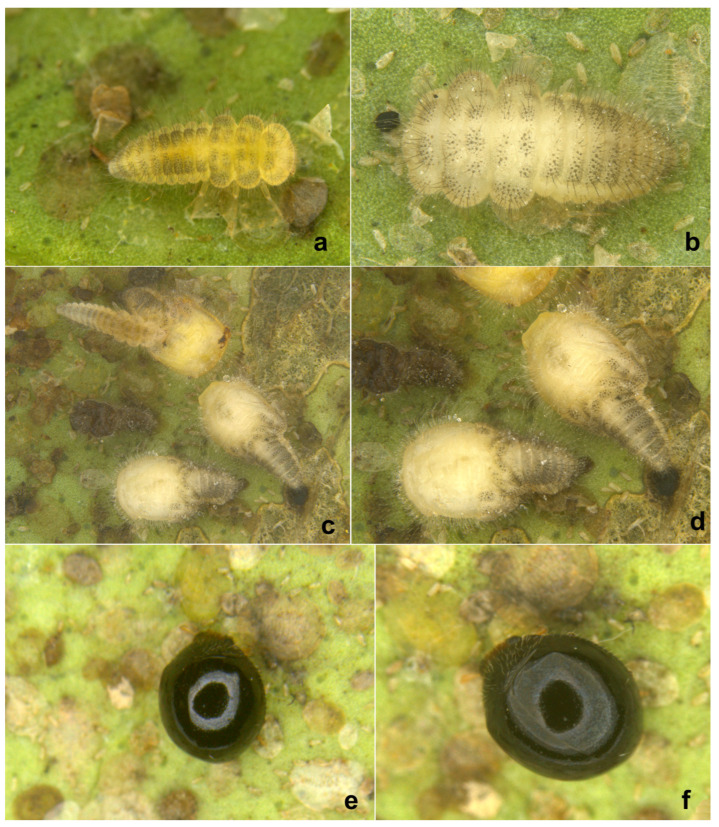
*Serangium chapini* (Kapur), life stages: (**a**,**b**) larva; (**c**,**d**) pupa; (**e**,**f**) adult.

**Figure 6:**
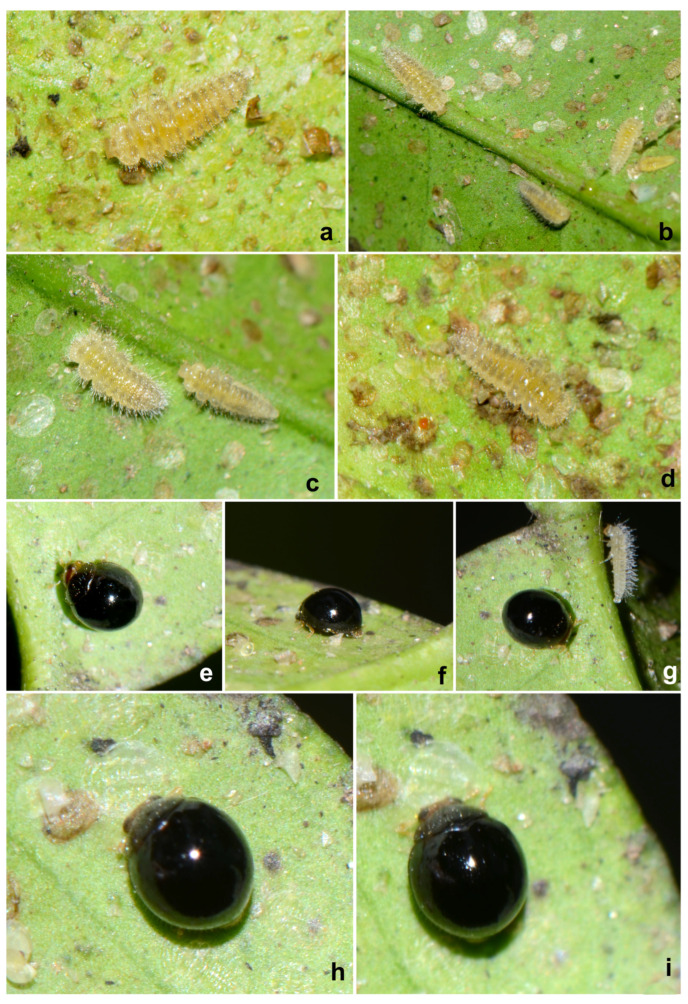
*Serangium chapini* (Kapur), life stages feeding on citrus whitefly: (**a**–**d**) larva; (**e**–**i**) adult.

***Serangium chapini* (Kapur)**

(Figure 3, Figure 4, Figure 5 and Figure 6).

*Catana chapini* Kapur, 1956: 189 [36]. Poorani, 2002: 360 [37]. Kovář, 2007: 569 [38].

*Serangium chapini*: Escalona and Ślipiński, 2012: 138 [1].

**Diagnosis.** Length: 1.70–2.00 mm; width: 1.50–1.80 mm. Form subhemispherical, strongly convex, slightly longer than broad. Dorsal side with head brown to castaneous, pronotum variable in color, piceous or castaneous to almost blackish except anterior and lateral margins paler; elytra piceous to black, shiny (Figure 3a and Figure 4a,c). Ventral side reddish brown to castaneous, occasionally median parts of metaventrite and abdominal ventrite 1 darker, piceous to blackish (Figure 4b,d). Head and pronotum with silvery white, sparse, semi-erect hairs. Elytra with long thin hairs on basal margin, discal area with sparse semi-erect hairs, lateral margins with distinct, suberect hairs. Antenna with eight antennomeres, terminal antennomere spatulate (Figure 3c). Abdominal postcoxal line incomplete (Figure 3d and Figure 4e). Male genitalia (Figure 3h–l and Figure 4h–l), female genitalia (Figure 3f and Figure 4f), and spermatheca (Figure 3g and Figure 4g), as illustrated.

**Immature stages.** The life stages of *S. chapini* feeding on citrus whitefly (*Dialeurodes citri* Ashmead) are illustrated in Figure 5 and Figure 6.

**Material examined.** INDIA: West Bengal: Cooch Behar, UBKV, without other data, two males, one female, five unsexed (ICAR-NRCB); INDIA: Uttarkhand, Almora, N 29°37′0″ E 79°40′0″, viii.2012, Ex. Citrus whitefly, J. Poorani, 25 ex. (ICAR-NBAIR).

**Distribution.** India (Uttarakhand; West Bengal).

**Prey/associated habitat.** All life stages illustrated here (Figure 5 and Figure 6) were collected in association with citrus whitefly (*Dialeurodes citri* (Ashmead)) (Aleyrodidae) from Uttarakhand. The type material was also collected on the same host [36].

**Seasonal occurrence.** May, August–September (label data).

**Notes.** The coloration of the head and pronotum in this species is variable from reddish brown to black. Kapur [36] described it from Jeolikot (Uttarakhand state) and provided only truncated illustrations of the male genitalia. For this study, specimens collected from Uttarakhand and West Bengal were examined.

**Figure 7:**
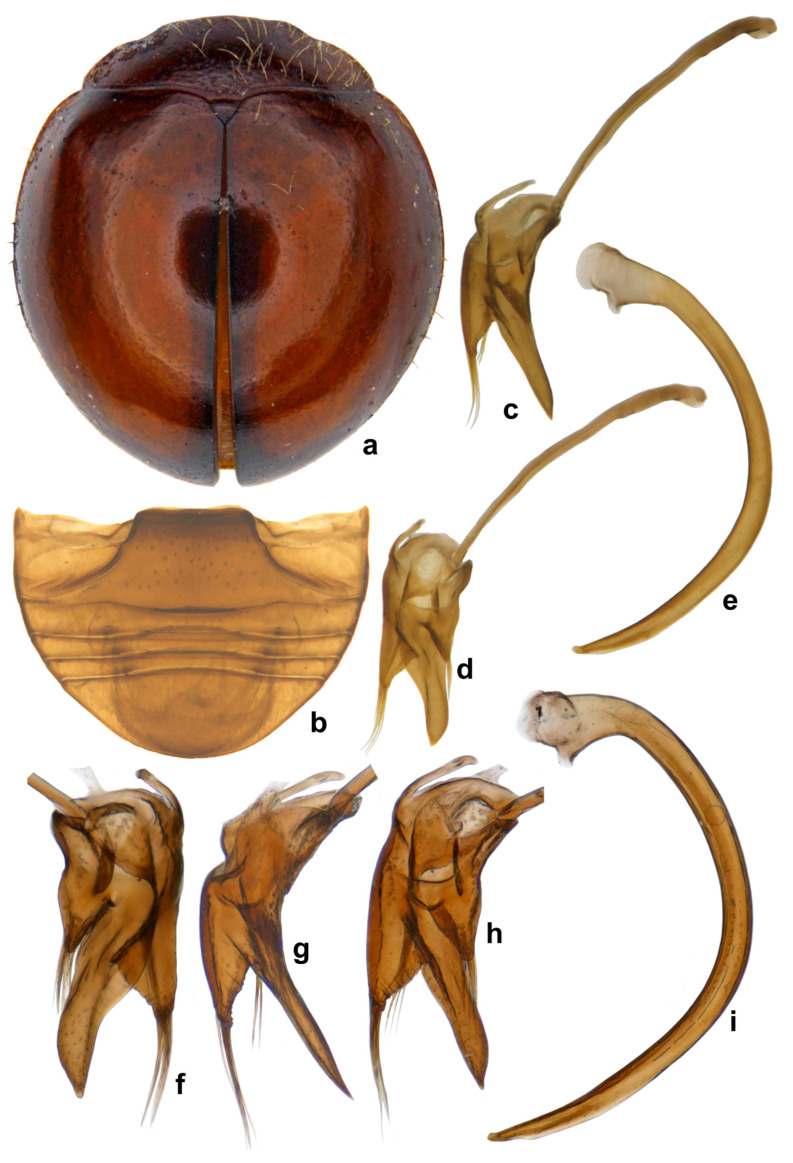
*Serangium clauseni* (Chapin): (**a**) dorsal view; (**b**) abdomen, male; (**c**–**i**) male genitalia, intraspecific variations: (**c**,**g**) tegmen, lateral view; (**d**,**h**) tegmen, outer view; (**f**) tegmen, ventral view; (**e**,**i**) penis.

**Figure 8:**
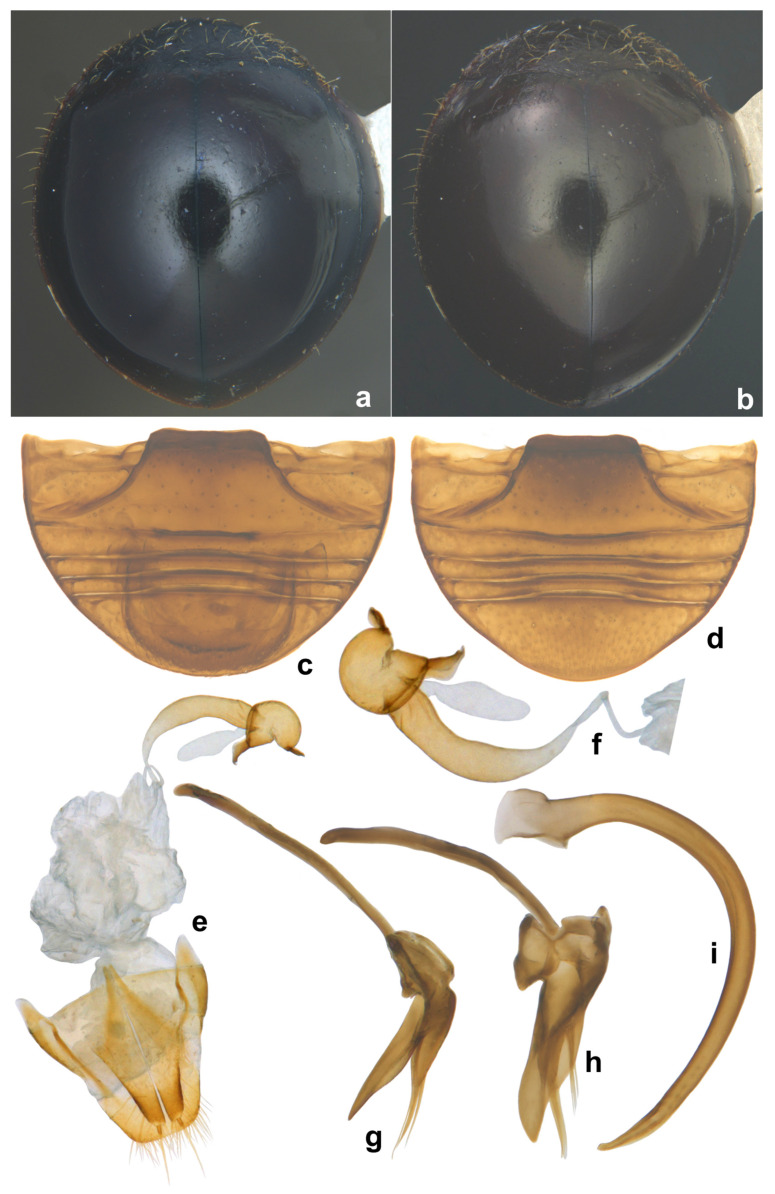
*Serangium* nr. *clauseni* (Chapin): (**a**,**b**) dorsal view; (**c**) abdomen, male; (**d**) abdomen, female; (**e**) female genitalia; (**f**) spermatheca; (**g**–**i**) male genitalia: (**g**) tegmen, lateral view; (**h**) tegmen, ventral view; (**i**) penis.

***Serangium clauseni* (Chapin)**

(Figure 7 and Figure 8).

*Catana clauseni* Chapin, 1940: 267 [29]; Miyatake, 1961: 139 [24]; Ren et al., 2009: 36 [39].

*Serangium clauseni*: Ślipiński and Burckhardt, 2006: 50 [28]. Wang et al. 2011: 40 [33]; Escalona and Ślipiński, 2012: 138 [1].

**Diagnosis.** Length: 1.95–2.24 mm; width: 1.66–1.98 mm. Form broad oval, dorsum strongly convex and hemispherical, shiny and glabrous. Head orange to brown, pronotum reddish to dark brown, anterior corners paler. Scutellar shield reddish to dark brown; elytra with discal area reddish brown-castaneous, margins much darker brown (Figure 7a). Underside yellowish to reddish brown, except prosternum darker brown. Legs yellowish brown. Abdominal postcoxal line (Figure 7b) incomplete. Male genitalia (Figure 7c–i) as illustrated, tegmen asymmetrical, penis guide narrow and distinctly pointed apically in lateral view (Figure 7c,g), tongue-shaped in ventral view (Figure 7f) and dorsal view (Figure 7d,h), parameres triangular with long apical hairs (Figure 7f–h); penis moderately stout, strongly curved, and narrowed towards apex (Figure 7e,i).

**Material examined.** INDIA: Assam: Jorhat, 25.10.11, Host: Mulberry, Coll. Y. Debaraj, two males, two unsexed (NRCB).

**Distribution.** India (Assam); China; Indonesia; Malaysia; introduced into Cuba and established [29]. Chapin [29] mentioned that the original releases of “*Catana clauseni*” were made in Cuba in 1930 and were “firmly established on the island” based on collections in 1938. This was also cited later by Gordon [14]. Chapin [29] also mentioned “the species has been planted at Nassau, Bahamas, but no recoveries have so far been made from that locality”. However, Wang et al. [33] mentioned only China as its distribution range.

**Prey/associated habitat.** The specimens examined in this study were collected on mulberry (label data). It was introduced to Cuba from Indonesia to control the citrus blackfly, *Aleurocanthus woglumi* Ashby [14,29].

**Notes.** Wang et al. [33] treated *S. clauseni* in their revision of Chinese *Serangium*. *Serangium clauseni* is very close to *S. japonicum* and the latter differs from it by its almost fully black dorsal coloration and the genitalia. Two specimens (one male and one female) examined in this work from Andaman Islands (Figure 8a,b) have reddish heads but the elytra are black as in *S. japonicum.* The female genitalia (Figure 8e) match those of *S. clauseni* but the male genitalia is slightly different and are closer to *S. japonicum* (Figure 8g–i) (Material examined: India: Andaman and Nicobar, Mount Harriet National Park, 11°42′57.96″ N 92°44′2.04″ E, 25.ii.2016, S.K. Rajeshwari, one male, one female).

**Figure 9:**
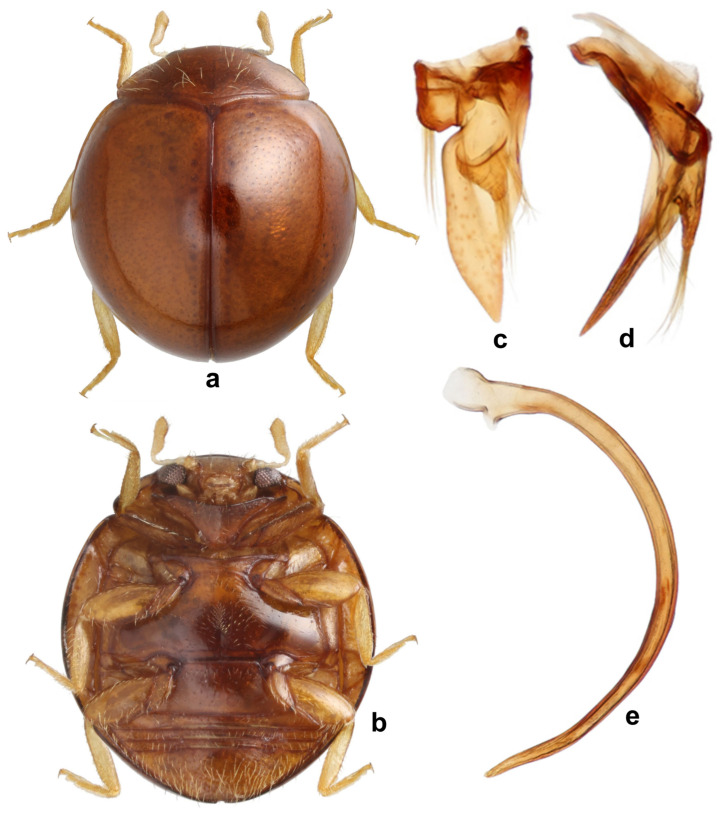
*Serangium montazerii* (Fürsch): (**a**) dorsal view; (**b**) ventral view; (**c**–**e**) male genitalia: (**c**) tegmen, ventral view; (**d**) tegmen, lateral view; (**e**) penis. (Photo from: www.zin.ru\Animalia\Coleoptera. Author—K.V. Makarov (accessed on 31 October 2024)).

**Figure 10:**
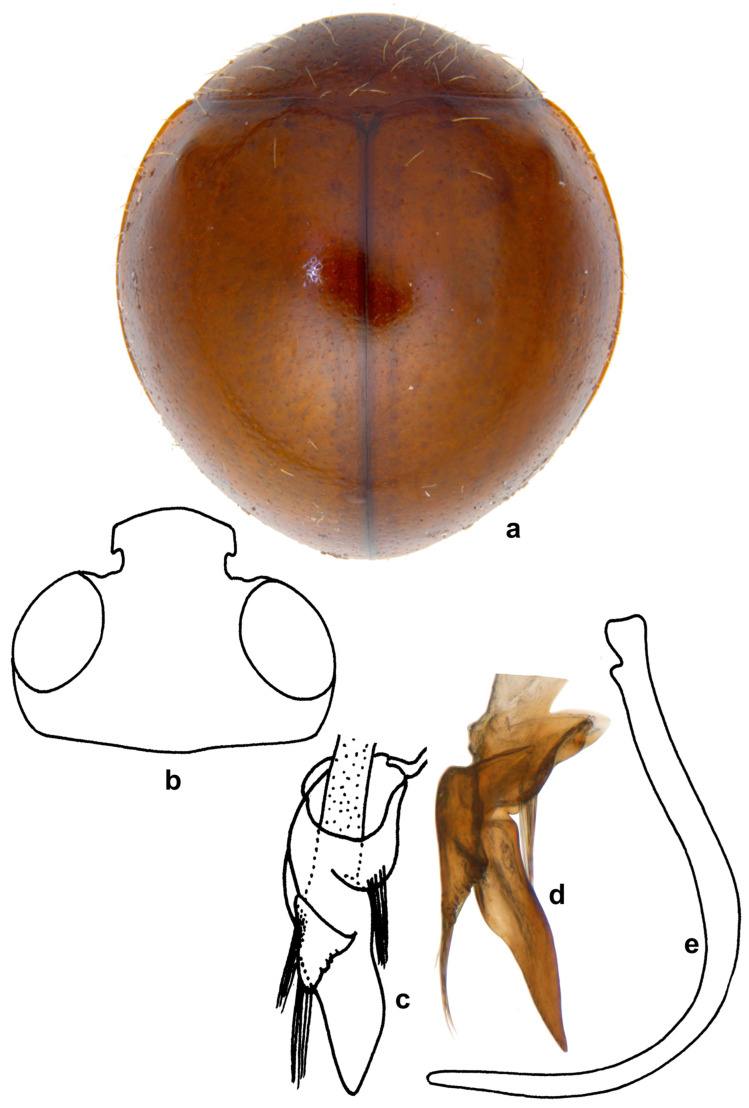
*Serangium montazerii* (Fürsch): (**a**) dorsal view; (**b**) head; (**c**–**e**) male genitalia: (**c**) tegmen, ventral view; (**d**) tegmen, lateral view; (**e**) penis.

***Serangium montazerii* Fürsch**

(Figure 9 and Figure 10).

*Serangium montazerii* Fürsch, 1995: 20 [40].

*Serangium parcesetosum* sensu Timofeyeva and Hoang, 1978: 302 [41].

*Serangium montazerii*: Booth and Polaszek, 1996: 72 [42]; Poorani, 2002: 361 [37].–Kovář, 2007: 569 [38].–Escalona and Ślipiński, 2012: 139 [1].

**Diagnosis.** Length: 1.92–2.10 mm; width: 1.62–1.75 mm. Form (Figure 9a and Figure 10a) short oval to almost circular, strongly convex, with sparse silvery white pubescence on pronotum and anterior and lateral margins of elytra. Uniformly yellowish to orange/reddish brown on both sides, ventral side occasionally slightly darker (Figure 9b). Head with interocular distance more than twice the eye width (Figure 10b). Similar to *S. parcesetosum,* can be separated from the latter by the fine elytral punctation, a little more noticeable than that of *S. parcesetosum* and slightly smaller, more widely separated eyes. Male genitalia (Figure 9c–e and Figure 10c–e) diagnostic, tegmen with right paramere rounded and left paramere triangular, as illustrated.

**Material examined.** Paratype male: Specimen on cardpoint, abdomen glued to the same card, “Paratype (yellow bordered circular label)/IRAN-Mazandran Prov. On citrus, leg. Montazeri 94/Paratype Serangium montazerii Fürsch, 1994 (rectangular, red label)/ex. Coll. H. Fürsch/genitalia in glass vial/Pres. By Int. Inst. Ent. BMNH [E] 1996-80” (BMNH).

**Distribution.** India: Appears to be restricted to northern and northwestern regions (Himachal Pradesh; Jammu and Kashmir; Uttarakhand; Uttar Pradesh); Pakistan; Middle East; Iran; Syria; Jordan; Turkey; Introduced in Israel, parts of Europe (Caucasus: Azerbaijan, Abkhazia, Georgia, south Russia; and Corsica, France); now considered an alien invasive in Israel, France, Georgia, Russia (Sochi) [43]. Migeon and Arabuli [44] rediscovered it in Georgia. Massimino Cocuzza et al. [45] recorded it in Italy (Sicily).

**Prey/associated habitat.** Hemiptera: Aleyrodidae: *Aleurocanthus* spp., *Aleurocanthus spiniferus* (Quaintance) [45], *Dialeurodes citri* (Ashmead), *Trialeurodes vaporariorum* (Westwood), *Bemisia tabaci* (Gennadius), *B. argentifolii* Bellows and Perring. Collected on citrus (label data).

**Seasonal occurrence.** Collected during May, July, and October (Uttar Pradesh).

**Notes.** *Serangium haleemae* Afroze and Haider, 1999 [46], described from northern India, is most likely to be a synonym of *S. montazerii.* The holotype of this species is not traceable at Aligarh Muslim University, cited as the depository by Afroze and Haider [46]. But the illustration of the penis suggests that it is synonymous with *S. montazerii* though the other parts are not properly illustrated. Fürsch [40] described it from Iran and it appears to have a wide distribution from the Middle East to the Indian Subcontinent. Timofeyeva and Hoang [41] studied its morphology and biology as “*Catana parcesetosa*” and also described the larva and pupa. Booth and Polaszek [42] corrected this misidentification and clarified its status as *S. montazerii* with illustrations of the male genitalia. Poorani [9] provided brief notes on its presence in India and illustrated the male genitalia. Good illustrations of the Indian material could not be provided due to the paucity of sufficient material for study. The illustrations of *S. montazerii* from the Atlas of Beetles of Russia are reproduced here with due credit (https://www.zin.ru/animalia/coleoptera/rus/SERANMONT.htm, accessed on 30 October 2024). Vatansever et al. [47] developed mass culturing techniques for *S. montazerii*.

**Figure 11:**
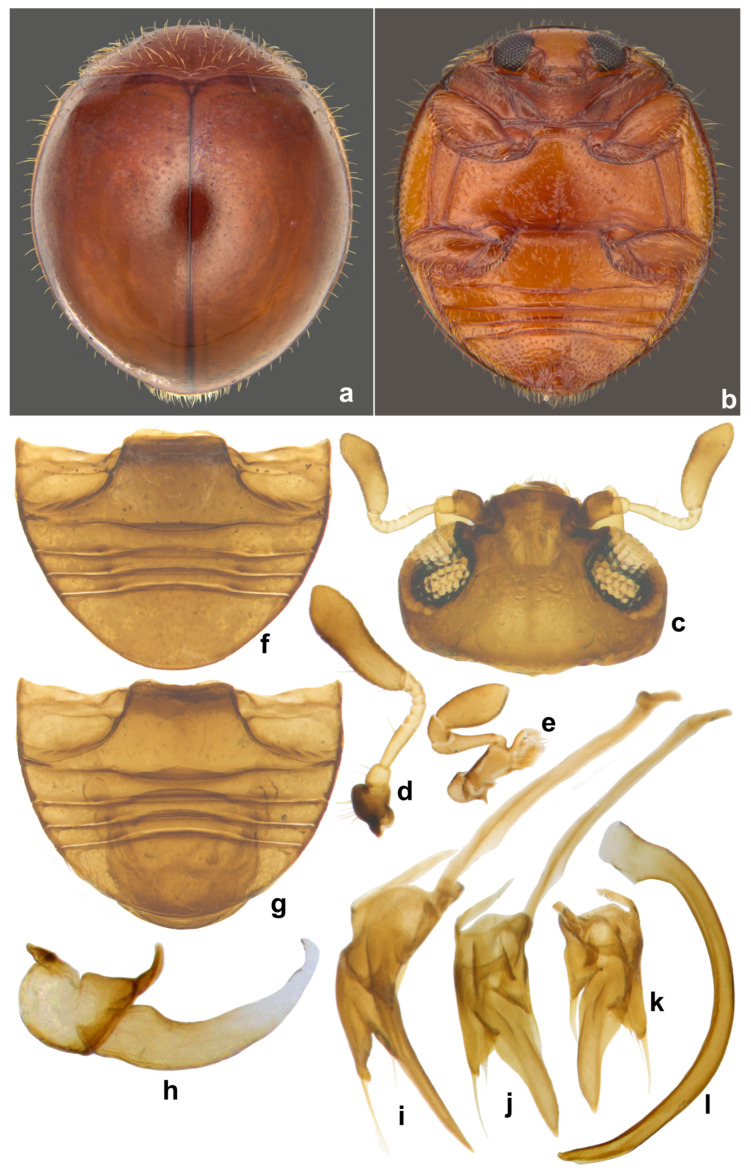
*Serangium parcesetosum* Sicard: (**a**) dorsal view; (**b**) ventral view; (**c**) head; (**d**) antenna; (**e**) maxilla; (**f**) abdomen, female; (**g**) abdomen, male; (**h**) spermatheca; (**i**–**l**) male genitalia: (**i**) tegmen, lateral view; (**j**) tegmen, ventral view; (**k**) tegmen, dorsal view; (**l**) penis.

**Figure 12:**
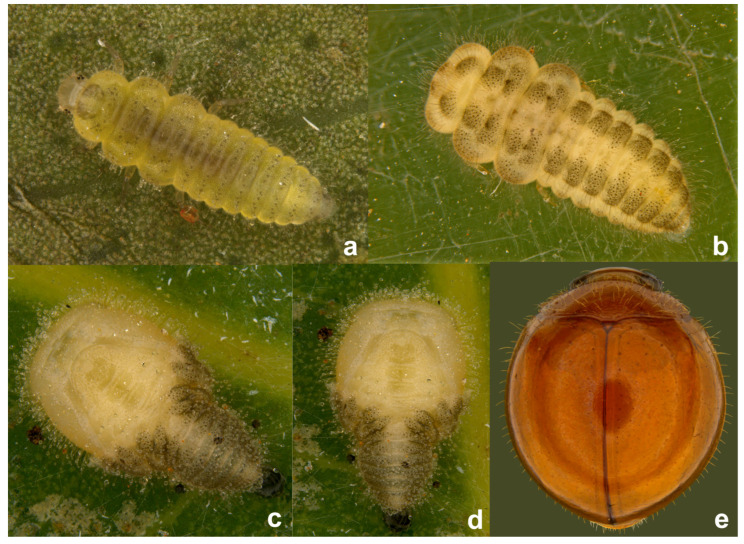
*Serangium parcesetosum* Sicard, life stages: (**a**) early stage larva; (**b**) mature larva; (**c**,**d**) pupa; (**e**) adult.

**Figure 13:**
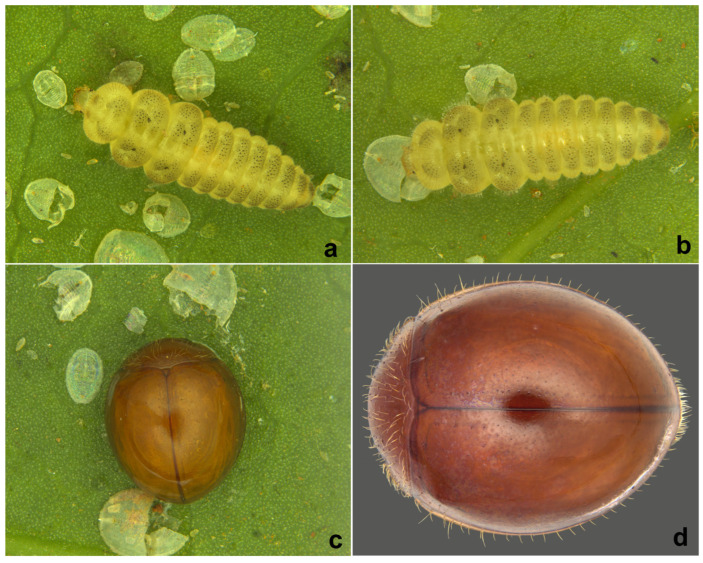
*Serangium parcesetosum* Sicard, life stages: (**a**,**b**) larva; (**c**) adult in whitefly colony; (**d**) adult.

***Serangium parcesetosum* Sicard**

(Figure 11, Figure 12 and Figure 13).

*Serangium parcesetosum* Sicard, 1929: 184 [48].

*Serangium parcesetosum*: Korschefsky, 1931: 218 [49]; Booth and Polaszek, 1996: 72 [42] (lectotype designation); Poorani, 2002: 361 [37]; Kovář, 2007: 569 [38].

*Catana parcesetosa*: Chapin, 1940: 268 [29].–Kapur, 1956: 189,193 [36].

**Diagnosis.** Length: 2.04–2.16 mm; width: 1.40–1.80 mm. Form hemispherical, only slightly longer than broad, strongly convex, dorsal side shiny with sparse, erect, thin silvery-white hairs on pronotum, anterior and lateral margins of elytra (Figure 11a and Figure 12e). Coloration uniform reddish brown or yellowish brown on both sides; a row of dark reddish brown spots, not amounting to true punctures, often present on either side of suture. Compound eyes large, coarsely faceted, interocular distance at its narrowest slightly more than or equal to 2x eye width (Figure 11c). Male genitalia (Figure 11i–l) asymmetrical, both parameres triangular.

Very similar to *S. montazerii* in general appearance, can be differentiated mainly by larger and less broadly separated eyes (interocular distance slightly more than twice the eye width in *S. montazerii*) and the male genitalia with tegmen having both lobes triangular. Further, *S. montazerii* is paler and elytral punctations are finer and more prominent than those of *S. parcesetosum* [42].

**Immature stages.** Larva (Figure 12a,b and Figure 13a,b) pale greyish with a greenish tinge, spindle-shaped, thoracic segments 2 and 3 distinctly wider, abdominal segments gradually narrowed towards apex in the posterior half. Pupa (Figure 12c,d) distinctly broader in the anterior half, much narrowed apically.

**Material examined.** “Type (red bordered circular label)/S. INDIA, COIMBATORE, Grub feeding on castor Aleurodes, 20.iv.28, T.K.V. Coll./Pres. By Imp. Bur. Ent. Brit. Mus. 1928-490/52/28/No.4/Serangium parcesetosum Sic., Type”, genitalia in glass vial, pinned with the specimen (BMNH). India: Tamil Nadu: Posampatti, Trichy Dt., N 10°46′51.17″ E 078°34′54.05″, 26.ix.17, 4.x.17, 26.x.17, on jasmine, R. Thanigairaj, 14 ex (ICAR-NRCB); India: Tamil Nadu: Podavur, NRCB farm, N 10°47′20.16″ E 078°34′29.88″, 20.iii.2011, on citrus, R. Thanigairaj, 1 ex; India: Tamil Nadu: Podavur, NRCB farm, N 10°47′20.16″ E 078°34′29.88″, 6.v.2022, 21.v.2022, and 23.v.2022, 4.vi.2022, on guava, R. Thanigairaj, 39 ex (ICAR-NRCB); Several specimens received for identification, without label data.

**Distribution.** India: Common in central and peninsular region, especially southern states (Bihar; Delhi; Gujarat; Haryana; Karnataka; Kerala; Gujarat; Maharashtra; Tamil Nadu; Uttar Pradesh); Thailand; Introduced into the USA, Israel [50], France, and Turkey; present in Uganda [51]. Introduced deliberately or accidentally into many countries, complete distribution not known.

**Prey/associated habitat.** Hemiptera: Aleyrodidae: *Aleurocanthus arecae* David, *Aleurocanthus woglumi* Ashby, *Aleurocanthus* sp., *Aleurodicus dispersus* Russell, *Aleurolobus barodensis* (Maskell), *Bemisia tabaci* (Gennadius), *Trialeurodes ricini* (Misra). Coccoidea: *Coccus hesperidum* Linnaeus, *Hsuia* sp., *Ferrisia virgata* (Cockerell).

Collected in association with whiteflies infesting cotton, castor, coconut, guava, *Jasminum* sp., *Syzygium cumini*, sunnhemp, sugarcane, and grasses (label data); Collected on eggplant, urd bean, mungbean, cotton, soybean, Indian bean, and rice bean [52]; predatory on *Paraleyrodes minei* Iaccarino and other coconut whiteflies, *Bemisia afer* (Priesner and Hosny), *Parabemisia myricae* (Kuwana) [53].

Introduced into the USA and Turkey for controlling *Bemisia tabaci* and the silverleaf whitefly, *Bemisia argentifolii* Bellows and Perring [2].

**Seasonal occurrence.** Collected during March–June and August–October in different parts of peninsular India; July–October in Haryana [52].

**Notes.** This is the most commonly collected species of *Serangium* in peninsular India. It was originally described from Coimbatore (Tamil Nadu, South India) and Booth and Polaszek [42] designated a lectotype for it. References to “*Cryptognatha flavescens*” from the Indian region in old literature [54,55], and Lefroy’s [56] illustrations of the life stages of “*Clanis soror*” are most likely to be based on either *S. parcesetosum* or *S. montazerii*. The species mentioned by Lefroy [56] and Misra [57] as ‘*Clanis soror* Weise’ is also a *Serangium*. Chapin [19], Booth et al. [58], Booth and Polaszek [42], and Poorani [9] treated this species.

**Figure 14:**
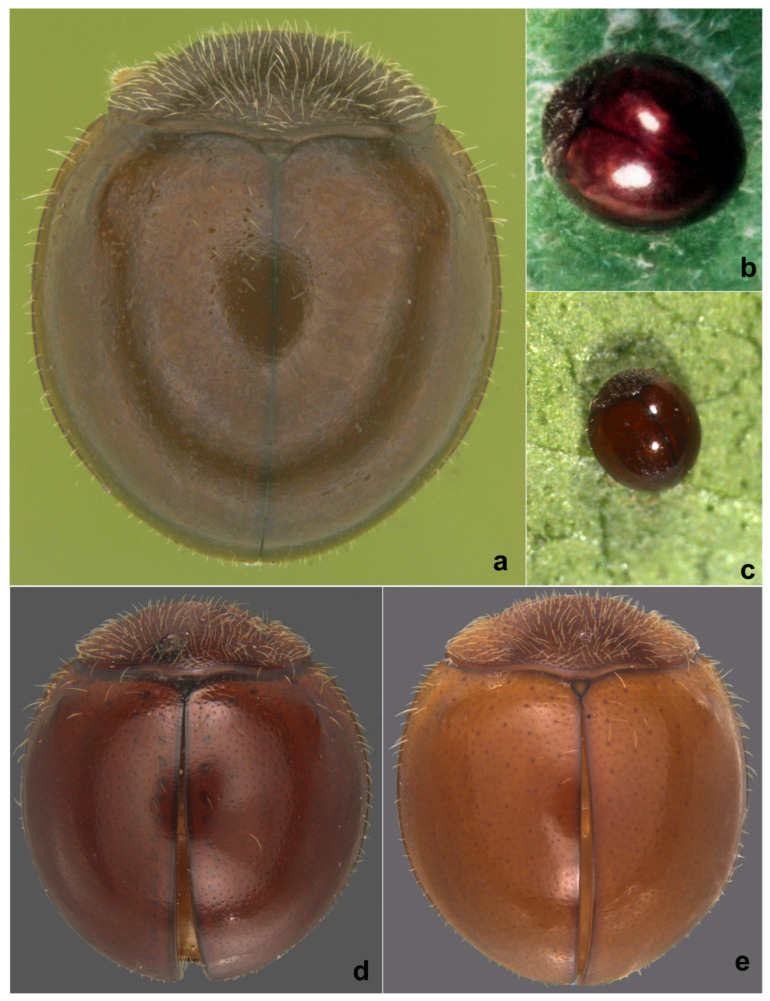
*Serangium serratum* Poorani: (**a**–**d**) nominate form; (**e**) paler variant.

**Figure 15:**
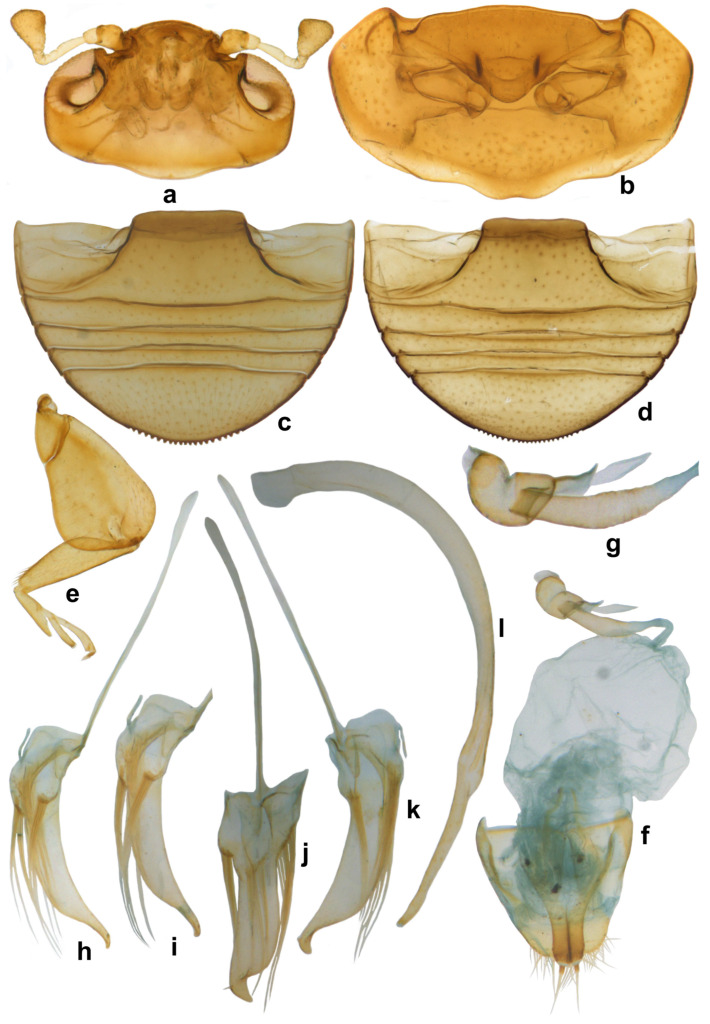
*Serangium serratum* Poorani: (**a**) head; (**b**) prosternum; (**c**) abdomen, female; (**d**) abdomen, male; (**e**) foreleg; (**f**) female genitalia; (**g**) spermatheca; (**h**–**l**) male genitalia: (**h**,**i**) tegmen, lateral view; (**j**,**k**) tegmen, ventral view; (**l**) penis.

***Serangium serratum* Poorani**

(Figure 14 and Figure 15).

*Serangium serratum* Poorani, 1999: 55 [9].–Poorani, 2002: 361 [37]; Escalona and Slipinski 2012: 139 [1].

**Diagnosis.** Length: 1.60–1.80 mm; width: 1.30–1.50 mm. Form small, hemispherical, highly convex; pubescence on pronotum uniform and dense, confined to lateral and basal margins of elytra. Dorsum of live specimens dark brown (Figure 14a–c), older specimens brown or much paler, reddish brown (Figure 14d,e), pronotum sometimes much darker than rest of body (Figure 14e). Mouthparts and legs yellowish brown and paler; mandibles and tarsal claws dark brown, elytral epipleura yellowish brown with a dark brown margin. Can be readily distinguished by the nine-segmented antenna with a pyriform club (Figure 15a), presence of a pair of dark brown basal ridges on prosternal process (Figure 15b) and serrate posterior margin of terminal abdominal ventrite in both sexes (Figure 15c,d). Male genitalia (Figure 15h–l) diagnostic, penis guide of tegmen apically strongly narrowed and produced, apical process obliquely and outwardly turned, parameres and middle of penis guide with very elongate hairs almost reaching apex of penis guide (Figure 15j); female genitalia (Figure 15f) and spermatheca (Figure 15g) as illustrated.

**Material examined.** Holotype male, “INDIA: Karnataka: Bangalore, 16.x.1997, Feeding on Heteropsylla cubana on Leucaena leucocephala, J. Poorani” (ICAR-NBAIR); Karnataka: Raichur, 2019, collected on citrus, Coll. Kiran, without other data, three males and one female (received for identification) (ICAR-NRCB).

**Distribution.** India (Karnataka; Tamil Nadu).

**Prey/associated habitat.** Hemiptera: Aleyrodidae: *Aleurodicus dispersus* Russell. Psyllidae: *Heteropsylla cubana* Crawford, *Diaphorina citri* Kuwayama. Collected on subabul, curry leaf, and citrus infested by psyllids (label data).

**Seasonal occurrence.** Collected during May; active in winter (November–January) in and around Bangalore.

**Notes.** Poorani [9] described it from Karnataka, southern India, as a predator of subabul psyllid, *Heteropsylla cubana* (Hemiptera: Psyllidae). Label data indicate it is most likely to be a psyllid predator as specimens examined for this study were collected on curry leaf and citrus infested by *Diaphorina citri* (detailed description, illustrations).

**Key to Indian species of *Serangium***

1. Posterior margin of last abdominal ventrite serrate (Figure 15c,d). Antennal club pear-shaped (Figure 1e and Figure 15a). Prosternal process with a pair of basal ridges (Figure 15b). Male genitalia (Figure 15h–l) with median lobe having an oblique, slightly upturned apex………………………………………………*serratum* Poorani

– Posterior margin of last abdominal ventrite not serrate (Figure 3d). Antennal club (Figure 1d) elongate, knife shaped. Prosternal process lacking basal ridges (Figure 1i). Male genitalia variable.………………………………………………2

2. Dorsal side uniform reddish brown………………………………………………3

– Dorsal side black or dark reddish brown with black margins………………………………………………4

3. Eyes large, not widely separated, interocular distance at its narrowest less than twice as wide as the eye (Figure 11c). Male genitalia with both parameres triangular (Figure 11i–k). Distributed in central and peninsular India………………………………………………*parcesetosum* Sicard

– Eyes smaller, more widely separated, interocular distance at its narrowest slightly more than twice as wide as the eye (Figure 10b). Male genitalia with right paramere broadly rounded (Figure 10c,d). Distributed in north and northwestern India………………………………………………*montazerii* Fürsch

4. Elytra black, head and pronotum dark reddish brown to black (Figure 3a and Figure 4a,c). Male genitalia (Figure 3h–l and Figure 4h–l) as illustrated………………………………………………*chapini* (Kapur)

– Elytra reddish brown with black margins (Figure 7a). Male genitalia (Figure 7b–e) as illustrated………………………………………………*clauseni* (Chapin)

### 3.3. Tribe Microweiseini

**Diagnosis.** Minute in size (<1 mm long), form elongate oval with dense dorsal pubescence. Head with clypeus anteriorly produced forward; eyes small, coarsely faceted; submentum distinctly narrower than the mentum; mentum deeply emarginate apically; maxillary cardo broad and clearly visible externally. Antenna with 10–11 antennomeres and a prominent, 2–3 segmented club. Pronotum with a distinct line or ridge separating anterolateral corners from rest of pronotum.

**Distribution.** Most of the genera and species of Microweiseini occur in the New World. Also known from South Africa, the Middle East, the Mediterranean region, and Australia [1].

The genus *Scymnomorphus* is represented in the Indian mainland with one species, *Scymnomorphus popei* (Vazirani) [16]. One new species, *Scymnomorphus ochraceus* sp. n., from Kerala, southern India, is described here. Escalona and Ślipiński [1] included Pakistan in the distribution range of the genus *Paracoelopterus* Normand, but not much information is available on this record.

***Scymnomorphus* Weise**

*Scymnomorphus* Weise, 1897: 303 [21]. Type species: *Scymnomorphus rotundatus* Weise, 1897 [21], designated by Pope, 1962: 628 [10]. Ślipiński and Tomaszewska, 2005: 380 [59].–Fürsch, 2006: 116 [60]. Escalona and Ślipiński, 2012: 152 [1].

*Scotoscymnus* Weise, 1901: 458 (unnecessary replacement name) [61]. Gordon, 1977: 189 [12]; Fürsch, 1985: 283 [62]; Miyatake, 1994: 235 [25].

*Sukunahikona* Kamiya, 1960: 23 [63]. Type species: *Sukunahikona japonica* Kamiya, 1960 [63], by original designation. Synonymized by Fürsch, 1985: 283 [62].

*Hikonasukuna* Sasaji, 1967: 4 [32]. Type species: *Hikonasukuna monticola* Sasaji, 1967 [32], by original designation. Synonymized by Escalona and Ślipiński, 2012: 153 [1].

*Orculus* Sicard, 1931: 233 [64]. Type species: *Orculus castaneus* Sicard, 1931 [64], by monotypy. Synonymized by Escalona and Ślipiński, 2012: 153 [1].

**Figure 16:**
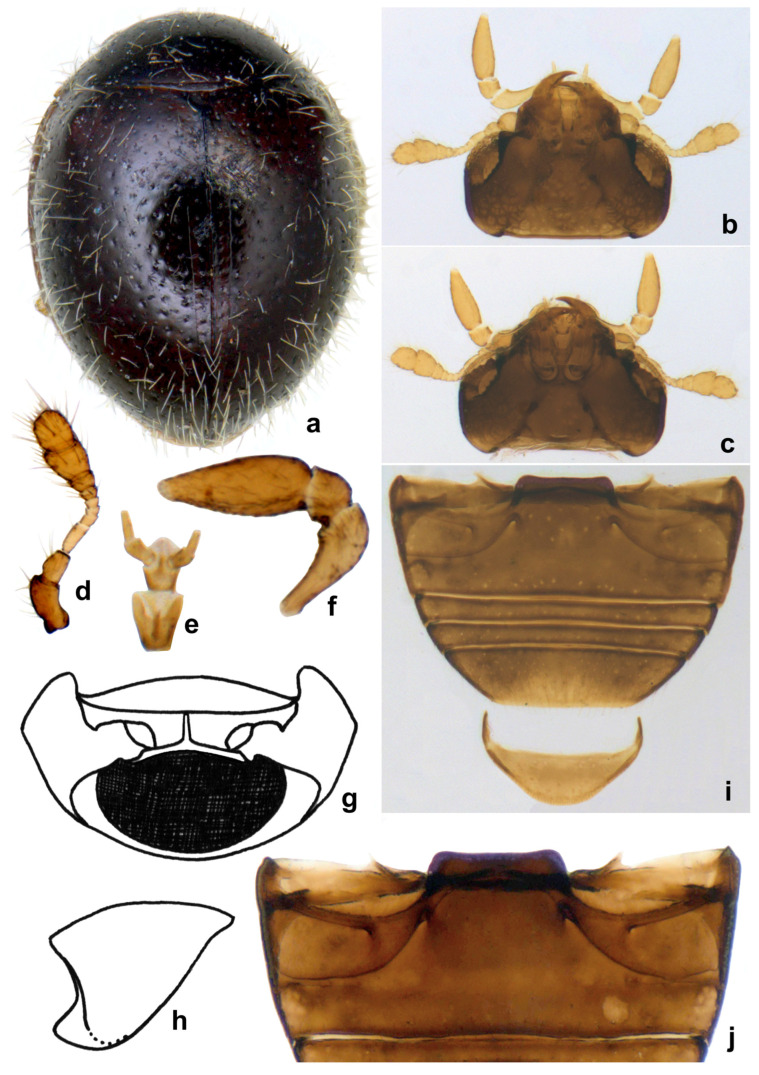
*Scymnomorphus popei* (Vazirani): (**a**) dorsal view; (**b**) head, dorsal view; (**c**) head, ventral view; (**d**) antenna; (**e**) labium; (**f**) terminal maxillary palpomere; (**g**) prosternum; (**h**) pronotum, lateral view; (**i**) abdomen, female; (**j**) abdominal postcoxal line.

**Figure 17:**
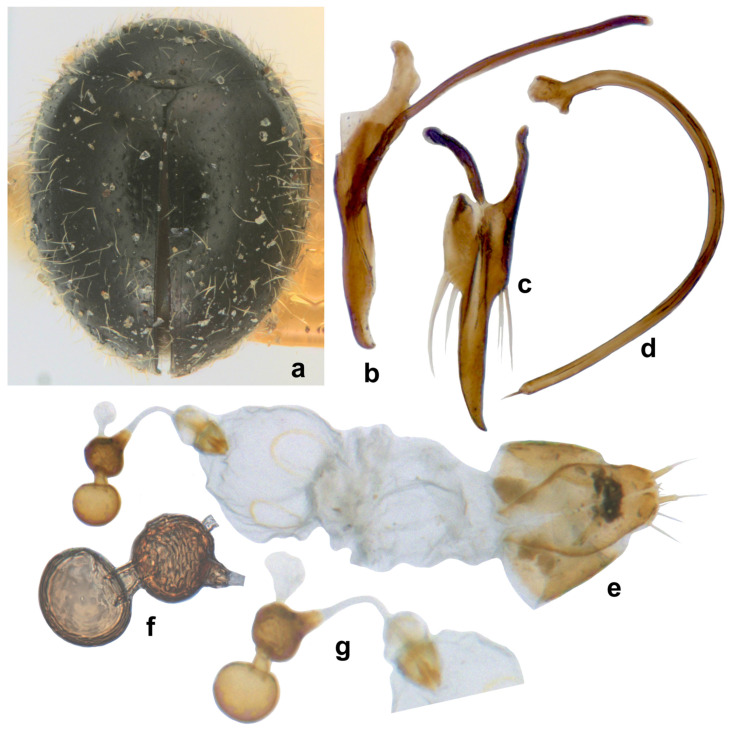
*Scymnomorphus popei* (Vazirani): (**a**) dorsal view; (**b**–**d**) male genitalia: (**b**) tegmen, lateral view; (**c**) tegmen, ventral view; (**d**) penis; (**e**) female genitalia; (**f**,**g**) spermatheca.

**Figure 18:**
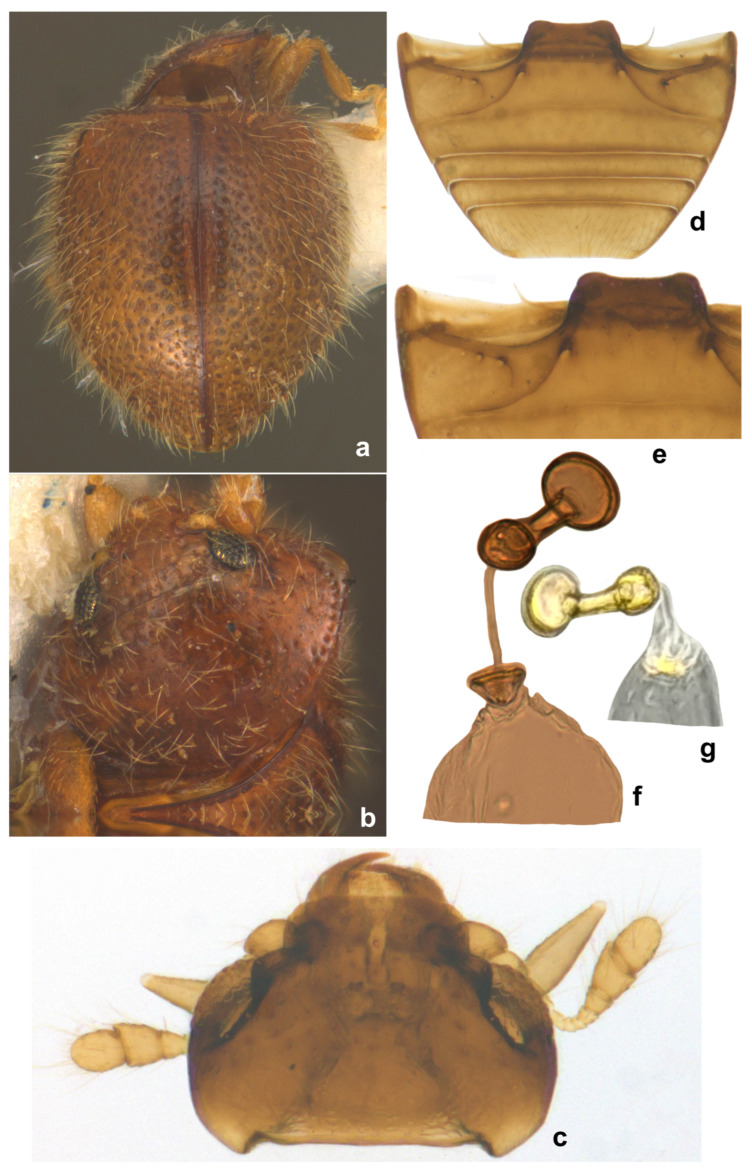
*Scymnomorphus ochraceus* Poorani, sp. n.: (**a**) dorsal view; (**b**) head and pronotum; (**c**) head; (**d**) abdomen, female; (**e**) abdominal postcoxal line; (**f**,**g**) spermatheca.

**Diagnosis.** Form very small to minute, elongate oval, distinctly convex, mostly winged. Dorsal surface with dense pubescence, usually consisting of a mixture of long and much shorter hairs (Figure 16a, Figure 17a and Figure 18a). Head with clypeus anteriorly produced forward, eyes small, coarsely faceted (Figure 16b and Figure 18c), usually with a characteristic cuticular pattern; submentum distinctly narrower than the mentum, mentum deeply emarginate apically (Figure 16c,e), maxillary cardo broad and clearly visible externally. Antenna with 9–10 antennomeres, club 2–3 segmented (Figure 16d). Pronotum with a distinct line or ridge separating anterolateral corners from rest of pronotum. Lateral sides of elytra with an epipleural carina originating at humeral angle and extending to the level of epipleura, parallel to lateral margin. Prosternal process very narrow and tubular, almost reduced to a narrow carina. Abdomen with six visible ventrites, ventrites 1 and 2 partially to fully fused. Abdominal postcoxal line incomplete or reaching lateral margin, usually with associated pits and pores. Male genitalia with penis guide asymmetrical, penis capsule distinct. Female genitalia with coxites elongate triangular, spermatheca dumbbell-shaped or bulbous on both ends with a median tubular constriction.

**Distribution.** This genus is pantropical and distributed in the Old and New World. Ślipiński and Tomaszewska [59] reviewed the Australian species and clarified the nomenclature of the genus.

**Biology.** Most of the known species are predatory on diaspine scales (Hemiptera: Diaspididae) [1].

**Indian species.** Only a single species, namely *Scymnomorphus popei* (Vazirani), predatory on coconut scales (*Chrysomphalus aonidum* (Linnaeus), and *Aspidiotus destructor* Signoret), is known at present in India from Gujarat and the Lakshadweep Islands. A second species is described here from Kerala, Southern India.

***Scymnomorphus popei*** **(Vazirani)**

(Figure 16 and Figure 17).

*Sukunahikona popei* Vazirani, 1982: 29 [16].

*Scotoscymnus popei*: Poorani, 2002: 368 [37].

*Scymnomorphus popei*: Escalona and Ślipiński, 2012: 153 [1].

**Diagnosis.** Length: 0.90–1.10 mm; width: 0.70–0.80 mm. Form (Figure 16a and Figure 17a) very small, elongate oval and narrowed towards elytral apex in posterior half; dorsum strongly convex, densely pubescent, elytra with long suberect hairs and shorter hairs intermixed. Dorsal and ventral side uniformly black or dark pitchy brown, elytra somewhat paler towards apices; mouthparts, antennae, and legs yellowish brown. Head transverse, with a characteristic reticulate–areolate pattern in the middle and on either side below eyes, more apparent in the posterior half (Figure 16b). Antenna (Figure 16b,d) with ten antennomeres, antennomeres 8–10 forming a distinct club. Terminal maxillary palpomere (Figure 16e) elongate conical to spindle-shaped. Pronotum with anterior corner separated by a carina (Figure 16g). Elytra with dual, deep punctures arranged in a characteristic pattern, one row of fine punctures arranged on either side of suture followed by a row of larger punctures, all large punctures marked by rings, with long hairs, somewhat irregular on disc and separated by 2–5 diameters, interspaces between large punctures having smaller punctures without halos and having shorter hairs (Figure 18a), lateral margins of elytra with large punctures forming 1–2 rows. Prosternal process (Figure 16f) narrow, tubular, and slender. Abdominal postcoxal lines (Figure 16h,i) incomplete with associated pores and pits; abdominal ventrites 1 and 2 almost completely fused medially, intersegmental lines visible only laterally (Figure 16h). Tarsi four-segmented. Male genitalia (Figure 17b–d) as illustrated. Female genitalia (Figure 17e) with coxites triangular, styli prominent, elongate; spermatheca (Figure 17f,g) composed of two bulbous lobes connected by a narrow median constriction, posterior bulb globular, anterior bulb having a dimpled surface and narrowly produced, sperm duct connected to the bursa with a prominent infundibulum.

**Material examined.** INDIA: Lakshadweep, Kavaratti, N 10°34′ E 72°37′, 7.iii.99, J. Poorani/On coconut infested with Aspidiotus destructor, Ceroplastes and mites, 1 ex; INDIA: Lakshadweep, Kalpeni, N 10°48′ E 73°38′, 6.iii.99, On coconut, J. Poorani, 4 ex. (ICAR-NBAIR).

**Distribution.** India (Gujarat; Lakshadweep Islands (Kalpeni, Kavaratti, Minicoy)).

**Prey/Associated habitat.** Diaspididae: *Aspidiotus destructor* Signoret infesting coconut (label data); *Chrysomphalus aonidum* (Linnaeus) on coconut (holotype label data); On coconut infested with *Aspidiotus destructor*, *Ceroplastes*, and mites (label data).

**Notes.** This is one of the smallest species of Coccinellidae in India, along with *Stethorus keralicus* Kapur. It was described as being from Gujarat, Western India. The validity of this species remains suspect as the male and female genitalia and other characters such as antenna and abdominal postcoxal lines are almost identical to those of *Scymnomorphus australis* (Chazeau, 1975) [12]. Chazeau [12] described *S. australis* from the west coast of Madagascar and also recorded it from the Comoros. However, the type material of *S. australis* (deposited at Muséum National d’Histoire Naturelle, Paris) could not be examined for confirmation.

The specimens collected from the Lakshadweep Islands (Minicoy, Kalpeni, Kavaratti) on coconut were studied in this work, and they fully match Vazirani’s description. There is a small spine-like projection on the inner side of the penis, a little below the penis capsule, which was not mentioned by Vazirani [16]. Vazirani [16] stated it was collected along with *Cybocephalus* sp. (Coleoptera: Cybocephalidae), another specific, commonly collected predator of scale insects on coconut.

***Scymnomorphus ochraceus*, sp. n.**

(Figure 18).

urn:lsid:zoobank.org:act:6454B957-253A-4911-A3A1-9D3D64D4B09B

**Etymology.** The specific epithet is a Latin adjective in reference to the yellowish-brown/ochraceous coloration of this species on both sides.

**Diagnosis.** It differs from *S. popei*, its sole Indian congener, by its slightly less elongate and broader outline, yellowish brown color, distinctly longer dorsal pubescence (Figure 18a), lateral and posterior margins of pronotum with a series of deep punctures arranged in a row (Figure 18b), and elytra with dual punctures, larger punctures marked by dark brown halos, and punctures partially arranged in rows. The female genitalia are also distinctive in having a differently shaped spermatheca (Figure 18f,g).

**Description.** Length: 0.90 mm; width: 0.75 mm. Form elongate oval, elytra narrowed in posterior half towards apices in posterior half; dorsum strongly convex, densely pubescent, elytral pubescence consisting of a mixture of long and shorter hairs (Figure 18a). Dorsal and ventral sides are uniformly yellowish brown (Figure 18b). Head (Figure 18c) transverse, with few somewhat irregularly spaced frontal punctures, sparsely distributed finer punctures below eyes, sides of eyes, middle and posterior one-fourth of the head with a characteristic water droplet-like pattern. Antenna with ten antennomeres, club three-segmented, terminal antennomere elongate oval; terminal maxillary palpomere elongate conical, distinctly narrowed apically (Figure 18c). Pronotum characteristic in having a series of punctures along the lateral and posterior margins (Figure 18b), punctures on disc dense, separated by 3–4 diameters. Elytra with dual, deep punctures arranged in a characteristic pattern, one row of fine punctures arranged on either side of suture followed by a row of larger punctures, all large punctures marked by dark brown rings, with long hairs and separated by 1–3 diameters, interspaces between large punctures having smaller punctures without halos and having shorter hairs (Figure 18a), lateral margins of elytra with large punctures forming 1–2 rows. Abdominal ventrites 1 and 2 incompletely and partially fused, intersegmental lines visible medially and laterally (Figure 18d); abdominal postcoxal lines incomplete, reaching the lateral margin of ventrite 1, with associated pores and pits (Figure 18d,e). Coxites triangular with elongate styli, spermatheca (Figure 18f,g) roughly dumbbell-shaped, posterior bulb larger than anterior, sperm duct connected to bursa by a distinct infundibulum (Figure 18f).

**Type material.** Holotype female: “INDIA: Kerala, Eravikulam N.P., 8.iii.2014, Shameem K Coll.” (fully dissected, in microvial) (ICAR-NBAIR).

**Distribution.** India (Kerala).

**Key to Indian species of *Scymnomorphus***

Both sides black to dark brown, elytral pubescence comprising a mixture of long and shorter hairs (Figure 16a and Figure 17a). Lateral and posterior margins of pronotum without a series of punctures. Spermatheca (Figure 17e–g) and male genitalia (Figure 17b–d) as illustrated. Distributed in Gujarat, Lakshadweep Islands………………………………………………*popei* (Vazirani)

–Both sides yellowish brown, elytral pubescence dual and distinctly longer (Figure 18a). Lateral and posterior margins of pronotum with a series of deep punctures arranged in a row (Figure 18b). Spermatheca (Figure 18f,g) as illustrated. Distributed in Kerala………………………………………………*ochraceus* Poorani, sp. n.

**An updated checklist of Microweiseinae of India**

**Family Coccinellidae Latreille, 1807**

**Subfamily Microweiseinae Leng, 1920**

**Tribe Serangiini Pope, 1962**

**Genus *Microserangium* Miyatake, 1961**

**1. *Microserangium brunneonigrum* Poorani, 2000**

Distribution: India: Tamil Nadu.

**Genus *Serangium* Blackburn, 1889**

**1. *Serangium chapini* (Kapur, 1956)**

Distribution. India: Uttarakhand; West Bengal.

**2. *Serangium clauseni* (Chapin, 1940)**

Distribution. India: Northeastern region (Assam); China; Indonesia (Sumatra); Malaysia; Cuba (introduced).

**3. *Serangium montazerii* Fürsch, 1995**

Distribution. India: Northern and northwestern regions (Jammu and Kashmir; Uttarakhand; Uttar Pradesh); Pakistan; Middle East; Introduced into Israel and Europe.

**4. *Serangium parcesetosum* Sicard, 1929**

Distribution: India: Widely distributed (Gujarat; Karnataka; Kerala; Lakshadweep Islands; Pondicherry; Maharashtra; Tamil Nadu); Thailand; Introduced into Turkey and the USA.

**5. *Serangium serratum* Poorani, 1999**

Distribution: India: Karnataka; Tamil Nadu.

**Tribe Microweiseini Leng, 1920**

**Genus *Scymnomorphus* Weise, 1897**

**1. *Scymnomorphus ochraceus* Poorani, sp. n.**

Distribution. India: Kerala.

**2. *Scymnomorphus popei* (Vazirani, 1982)**

Distribution. India: Gujarat; Lakshadweep Islands (Kavaratti, Kalpeni, Minicoy).

## 4. Discussion

Focused exploratory surveys and collections for Microweiseinae in the Indian region are lacking and only a handful of species are known at present, which probably represent a tiny fraction of the existing diversity. One species, *Serangium parcesetosum* is commonly collected on many host plants, including citrus, cotton, guava, jasmine, etc., and even grasses (personal observations). Both Indian species, *Serangium parcesetosum* and *S. montazerii*, are similar, and though both have been widely introduced in different parts of the world, considerable confusion exists about their diagnosis even today. Migeon and Arabuli [44] gave an account of the history of the introduction of *S. montazerii* in Europe under various names. Initial introductions of *S. montazerii* in parts of Europe in the 1970s (from North India (Raniket, Uttarakhand)) appear to be under the names “*S. parcesetosum/Catana parcesetosa*”. The original description of *S. montazerii* was published much later by Fürsch [40] based on Iranian specimens (Mazandran Province). Duverger [65] assigned all the introductions of *Serangium* in Western Europe (resulting from the first one introduced by Timofeyeva and Hoang [41] to *S. montazerii*. Even after this, Coutanceau [66] and Coutanceau and Malausa [67] assigned the French-introduced populations to *S. parcesetosum* based on specimens examined from Corsica but did not provide a comparative diagnostic account vis-à-vis *S. parcesetosum*. Migeon and Arabuli [44] stated that the material introduced in France (continental and Corsica) and Turkey came from the Georgian source studied by Timofeyeva and Hoang [41]. Diagnostic accounts of both *S. parcesetosum* and *S. montazerii* are given by Booth and Polaszek [42] and Poorani [9].

Though Serangiini are typically specific predators of whiteflies, other insects and mites may be used as prey. Among the Indian species, *S. serratum* has been recorded as a predator of psyllids (*Heteropsylla cubana, Diaphorina citri*) on subabul, curryleaf, and citrus. Biranvand et al. [68] reported *S. montazerii* as a predator of *Euphyllura olivina* (Costa) (Psyllidae) on citrus, olive, pomegranate and *Salvia*. Besides whiteflies, less-preferred prey like *Coccus hesperidum, Aphis gossypii*, *Frankliniella occidentalis*, and *Tetranychus urticae* may be used for feeding and reproduction by *S. parcesetosum* and *S. montazerii* (see Plant Pests of the Middle East, http://www.agri.huji.ac.il/mepests/enemy/Serangium_montazerii/, accessed on 25 October 2024).

Regarding Microweiseinae, specimens are hard to find in Indian repositories, probably due to their minute size and cryptic coloration. Only *Scymnomorphus popei* has been recorded in association with coconut scales in the West Coast region of India (Gujarat, Lakshadweep islands). As mentioned earlier, the validity of *S. popei* needs to be confirmed as it is almost identical to *S. australis,* described from the Indian Ocean islands of Madagascar and Comoros [12]. The spermatheca in *Scymnomorphus* appears diagnostic, but it is not illustrated for most known species.

More interestingly, utilization of the species of Microweiseinae of Indian origins, such as *S. parcesetosum* and *S. montazerii*, in applied biological control programs for the management of whiteflies has been extensive outside India. However, these predators have received very little attention in India [52,69], though they are commonly encountered on various agricultural and horticultural crops. Further, several alien invasive whiteflies from the New World have been reported in recent years from the Indian region, but their predators remain poorly studied, with parasitoids and entomofungal pathogens receiving greater attention in management programs [70,71]. Focused surveys targeting the predators of native and introduced whiteflies and diaspine scales in Indian agroecosystems are likely to lead to the identification of specific predators belonging to Serangiini and Microweiseini, including exotic species. For instance, *Delphastus pallidus* (LeConte, 1878), a New World genus and species of Serangiini, was recently recorded as a predator of cotton whitefly from the Punjab region of Pakistan [72]. However, information on the predators of cotton whitefly in the adjoining Indian border state of Punjab is lacking.

As emphasized by Szawaryn et al. [19], the greater diversity of Serangiini, particularly *Serangium*, in China and Australia, is most likely to be due to recent revisions and does not give a true picture of their diversity worldwide. As many as 15 species of *Serangium* [33,73], nine of *Microserangium* [30], and eight of *Scymnomorphus* [74] are currently known from China. In India, collections of Microweiseinae from the hotspots of Coccinellidae diversity, such as northeastern region (bordering China), are virtually non-existent in major museums. The known Coccinellidae fauna of the northeastern region has a lot in common with the Chinese fauna and the same is also likely to hold up well in the case of Microweiseinae. Hence, systematic collections and studies are required to unearth the potential and hidden diversity of Microweiseinae of the Indian region, particularly from hotspots, to enable their documentation and utilization in biological control in the long term.

## Data Availability

The original contributions presented in the study are included in the article, further inquiries can be directed to the corresponding author.
